# Editorial: The Emerging Role of Artificial Intelligence in Dermatology

**DOI:** 10.3389/fmed.2021.751649

**Published:** 2021-11-17

**Authors:** Farhan Mahmood, Solomon Bendayan, Feras M. Ghazawi, Ivan V. Litvinov

**Affiliations:** ^1^Faculty of Medicine, University of Ottawa, Ottawa, ON, Canada; ^2^Faculty of Medicine, McGill University, Montréal, QC, Canada; ^3^Division of Dermatology, University of Ottawa, Ottawa, ON, Canada; ^4^Division of Dermatology, McGill University, Montréal, QC, Canada

**Keywords:** artificial intelligence, machine learning, deep learning, dermatology, teledermatology, COVID-19

The use of artificial intelligence (AI) in dermatology is an emerging area of interest with several applications highlighted in the special topic, including the differentiation of benign and malignant pigmented lesions, improvement of diagnosis and management of psoriasis and other inflammatory diseases, assessing ulcer specifications, and gene expression profiling Gomolin et al.. Over the past decades, there has been a significant focus in analyzing and classifying data from skin lesions using machine learning (ML) models (1, 2). In this editorial, we highlight previous and recent applications of AI and its use during the coronavirus disease 2019 (COVID-19) pandemic.

It was previously illustrated that AI is able to distinguish between benign nevi vs. melanoma using individual pixels from dermatoscopic and non-dermatoscopic images (3–19). Jutzi et al. further assessed the attitudes of patients toward AI and demonstrated that most respondents supported the use of AI, particularly to help detect melanoma early at home. However, potential errors, poor/inconsistent image quality and insufficient data protection of AI still pose important barriers. Recently, ML and convolutional neural network (CNN) models that classify melanoma on histopathological or clinical images demonstrated ability to achieve exceedingly high sensitivities and diagnostic accuracies (20–24). ML models are also being trained using substantial data sets including more racially diverse data, making AI more accessible for use in remote and resource-limited healthcare settings (20, 25). There is also a rise in smartphone applications with classifying the risk of photographed lesions or detecting malignant/premalignant lesions on histopathological images (26, 27). One study assessed whether dermatoscopic or reflectance confocal microscopy (RCM) findings correlated with histologic diagnoses of melanocytic lesions with peripheral globules (28). They found that dermoscopy and RCM diagnosed 100% of melanomas and 84.21% of dysplastic nevi accurately. Dysplastic nevi and melanocytic lesions differed significantly based on the sizes and shapes of peripheral globules, and the signs of malignancy on RCM including pagetoid cells, non-edged papillae, atypical junctional thickenings, and atypical cells at the dermal-epidermal junction. A top-ranked computer algorithm has also been shown to classify images of melanomas, nevi, and seborrheic keratoses with a higher specificity than dermatologists (85.0 vs. 72.6%) (29). Thus, AI has the potential to classify skin images of melanoma and its benign mimickers with high accuracy.

However, despite the increased accuracy of diagnosing melanomas, clinicians or AI cannot reliably predict the oncologic transformation of nevi (30). This is due to the static nature of the nevi on clinical presentation, and the lack of data to train AI on the evolution of certain melanocytic nevi including dysplastic or spitzoid nevi. Further research is required to understand how AI can identify the progression of dysplastic nevi.

Gomolin et al. and Schäfer et al. both highlighted the use of AI in ulcer assessment. Schäfer et al. demonstrated that predictive values including low household income, older age, and comorbidities were associated with higher risks for diabetic foot ulcer (DFU) and amputation. However, ML models did not achieve reliable results when predicting the prognosis of DFUs or amputations using the predictive values. The authors highlighted the development of models that can detect wound progression of DFUs using predictive values as next steps Schäfer et al.. Recently, CNN models have emerged categorizing wound images based on their etiology (31) and are being implemented in smartphone application to detect DFU wounds with high inter- and intra-observer reliabilities compared to traditional measurements (32). Notably, the combination of clinical laboratory (e.g., glomerular filtration rate) data from health records and image features of patients with DFUs by a ML model predicted the healing of DFUs, with reliable accuracies (33). Polarized hyperspectral imaging and ML technology has also recently shown to be a potential avenue to characterize detailed pathological complications of ulcers (34).

Similarly, a review by Du et al. illustrated that ML has the potential to predict the clinical outcomes and prognosis of several dermatoses. Larger sample sizes of data enable ML algorithms to produce accurate outputs; however, this may be a limitation since national and international collaborations between registries are required to acquire large dermatologic data sets. The authors underscore the need for prospective clinical trials to validate the use of ML models to predict outcomes. Recently, a ML model accurately predicted the Dermatology Quality of Life Index for patients with psoriasis withdrawing from risankizumab (35), while similar ML technology was used to build a highly accurate biomarker predicting the progression of alopecia areata to alopecia totalis or alopecia universalis (36). A multicenter prospective open label pilot study was recently conducted treating psoriasis patients with secukinumab. A predictive model was developed using clinical attributes of patients, achieving an accuracy of 91.88% in predicting responders and non-responders (37).

A recent study by Showalter et al. (38) shed light onto the potential application of AI to inflammatory dermatology conditions. Showalter et al. aimed to determine the histologic and gene expression features of clinical improvement in early diffuse cutaneous systemic sclerosis (dcSSc) by evaluating skin biopsies from patients with dcSSc. They used support vector machine learning using scleroderma gene expression subset as classifiers and histology scores as inputs. In the samples with the highest Modified Rodnan Skin Score, alpha-smooth muscle actin (ASMA) was the highest and CD34 was the lowest, and these markers were the strongest predictors of gene expression subset. CD34 staining was the highest in the normal subset and ASMA was highest in the inflammatory subset. The CD34 and ASMA binarized scores also identified a 47-gene fibroblast polarization signature which decreased in patients with clinical improvement compared to those with no improvement. Systemic sclerosis histologic features have been shown to correlate with the Modified Rodnan Skin Score; however, this recent study highlights the potential to use dermal fibroblast polarization between aSMA and CD34 to describe clinical improvement for dcSSc.

Heckler et al. previously examined the effects of label noise on the performance of CNN models when classifying skin cancers. They found that ML models are highly sensitive to label noise, highlighting the need for biopsy-verified images to train models. Recently, a hybrid evolutionary optimization technique based on two swarm intelligence algorithms was alternatively proposed to overcome the premature local convergence of single conventional clustering algorithms when detecting and segmenting psoriasis lesions (39, 40). Global convergence of algorithms has demonstrated superiority over conventional clustering techniques (39, 40), revealing the potential to combine multiple algorithms to overcome the biases and restrictions of single models.

Importantly, the social distancing restrictions imposed by the COVID-19 pandemic fostered digital transformations in dermatology and sparked COVID-19-specific applications of AI and technology. For instance, several cutaneous manifestations are associated with COVID-19 including pernio-like and vesicular lesions (41); CNN models are being developed to detect these lesions in patients with Fitzpatrick I-VI skin, further optimizing contact-free healthcare (42, 43).

During the pandemic, there has also been an increased uptake of teledermatology worldwide to ensure patients and healthcare providers have access to dermatologists (44–46). Teledermatology enables patients from remote/rural areas and/or underserved populations to obtain access, while reducing wait times for dermatology referrals (47–49). Recently, ML models have been developed to assess the quality of photos submitted by patients to teledermatology consults—rejecting low quality and retaining high quality photographs (50). ML models are now being used to classify dermatology clinical images in the electronic health records to optimize access to these images for research purposes (51). Furthermore, recent studies have demonstrated that dermoscopy improves the diagnostic accuracy of teledermatology (52); thus, smartphone microscope applications employing CNN are now increasingly supplementing teledermatology consults (53). Furthermore, because several dermatoses are usually initially seen by primary care providers (PCP), an AI-based tool was developed to interpret images and their associated medical histories, which improved the diagnoses of dermatoses by PCPs in a teledermatology setting (54).

Social media has also become an important component of technology and dermatology during the COVID-19 pandemic. The use of social media in dermatology increased over the recent years and common uses for dermatologists include information dissemination, knowledge sharing, and networking (55–57). Thus, social media enables knowledge-exchange and interactivity during the pandemic—when access to dermatologists may be restricted.

Although there are several applications of AI in dermatology, there are certain barriers preventing its uptake. With respect to teledermatology, full-body skin examinations are difficult to perform virtually, thus, clinically significant lesions may be missed (58, 59). Several costs are also associated with the implementation of teledermatology (i.e., equipment costs, technological competencies, and staff training), while ensuring encryption to protect confidential medical data (60–62). Teledermatology may also be unavailable for individuals who do not have access to high quality internet or telecommunication devices with high quality cameras (63).

Several other barriers exist with regards to the implementation of AI in clinical dermatology, which have been extensively discussed by Gomolin et al. as well, including generalizability, standardization, and interpretability (3, 64–67). To summarize, several AI algorithms are trained using input data from limited populations, thus they may not be effective in patients from different settings or with unique phototypes. Universal quality standards for images used to train AI or used for dermoscopy are also lacking. Further, patients appreciate the traditional encounter with the physician and the accountability that it entails. Physicians alike, are resistant to accept that AI can diagnose and manage dermatoses more reliably than them. Finally, most original research studies in AI have not studied the applications in large-scale clinical trials. More high-quality clinical studies are needed to substantiate the use of AI in dermatology.

In conclusion, significant advances have been made to enable AI use in dermatology. Such advancements play essential roles in detecting, diagnosing, classifying, and prognosticating dermatoses. This field will continue to evolve with the focus on improving the diagnostic accuracy of ML models, determining the use of predictive models through prospective trials, and developing smartphone applications to optimize virtual healthcare.

## Author Contributions

All authors listed have made a substantial, direct and intellectual contribution to the work, and approved it for publication.

## Conflict of Interest

The authors declare that the research was conducted in the absence of any commercial or financial relationships that could be construed as a potential conflict of interest.

## Publisher's Note

All claims expressed in this article are solely those of the authors and do not necessarily represent those of their affiliated organizations, or those of the publisher, the editors and the reviewers. Any product that may be evaluated in this article, or claim that may be made by its manufacturer, is not guaranteed or endorsed by the publisher.
